# Supplementary material for: An Ensemble Docking Approach for Analyzing and Designing Aptamer Heterodimers Targeting VEGF165
Source: Int J Mol Sci. 2024 Apr 5;25(7):4066. doi: 10.3390/ijms25074066 (PMC11012306; doi:10.3390/ijms25074066)
Supplement: Supplementary file 1 [file ijms-25-04066-s001.zip › ijms-2941615-supplementary.pdf]

Supporting Information for

An ensemble docking approach for analyzing and designing aptamer heterodimers targeting $VEGF_{165}$

Yeon Ju Go [1,2], Mahroof Kalathingal [1,*] and Young Min Rhee [1,*]

[1] Department of Chemistry, Korea Advanced Institute of Science and Technology (KAIST), Daejeon 34141, Republic of Korea

[2] Department of Chemistry, Pohang University of Science and Technology (POSTECH), Pohang 37673, Republic of Korea

Supporting Figures

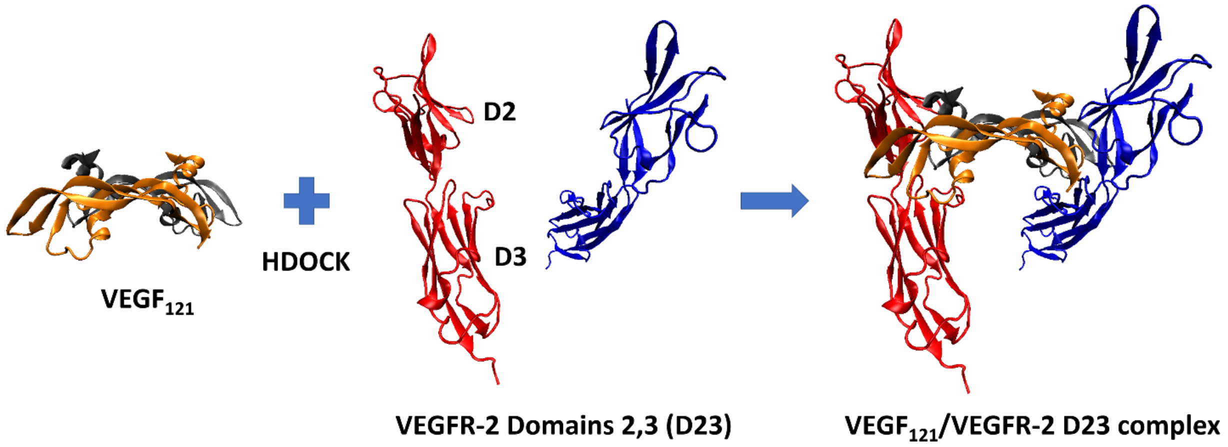

Figure S1. Schematic illustration of the testing process of the HDOCK performance. $VEGF_{121}$ and VEGFR-2 D23 were docked, and the resulting pose was cross-checked against the X-ray crystal structure of the VEGF-A/VEGFR-2 D23 complex. Indeed, they showed a good agreement. The two monomers of $VEGF_{121}$ are shown in orange and gray, while those of VEGFR-2 are shown in red and blue.

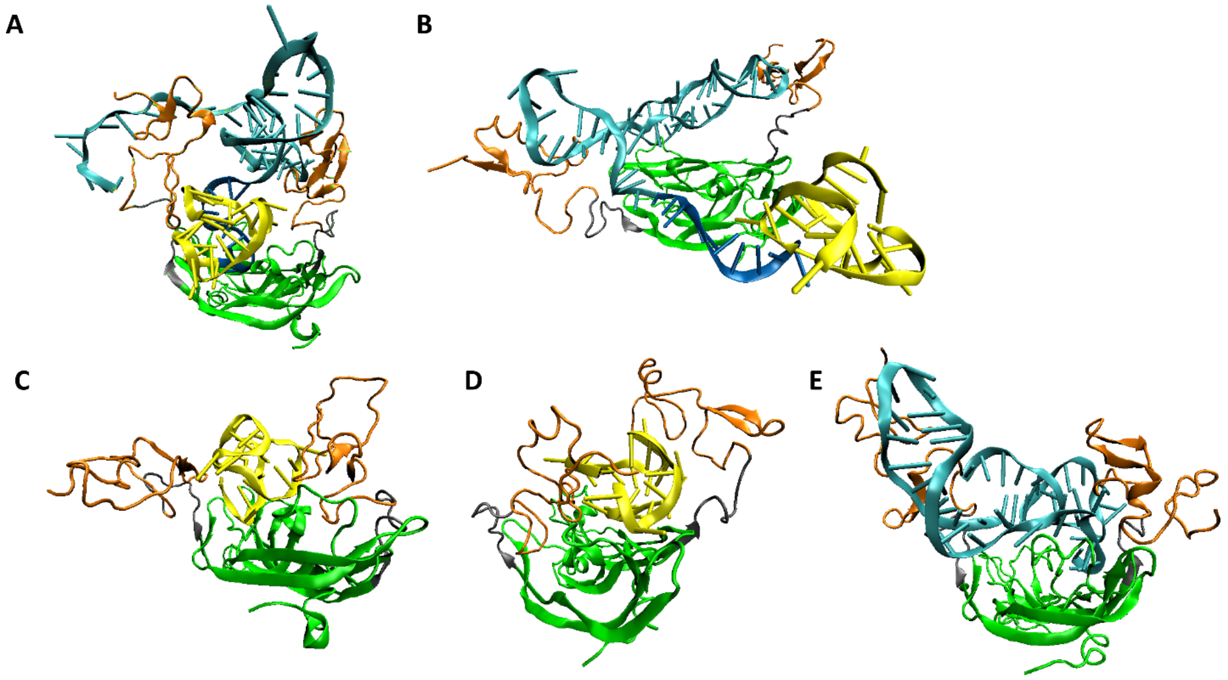

Figure S2. Representative structures of the sandwich poses for (A) $VEGF_{165}$/RNV66:del5-1, (B) $VEGF_{165}$/V7t1:del5-1, (C) $VEGF_{165}$/RNV66, (D) $VEGF_{165}$/V7t1, and (E) $VEGF_{165}$/del5-1. For the aptamers, RNV66 and V7t1 are shown in yellow while del5-1 is in cyan, with the linker in blue. For $VEGF_{165}$, RBD is in green while HBD is in orange, with the linker in gray.

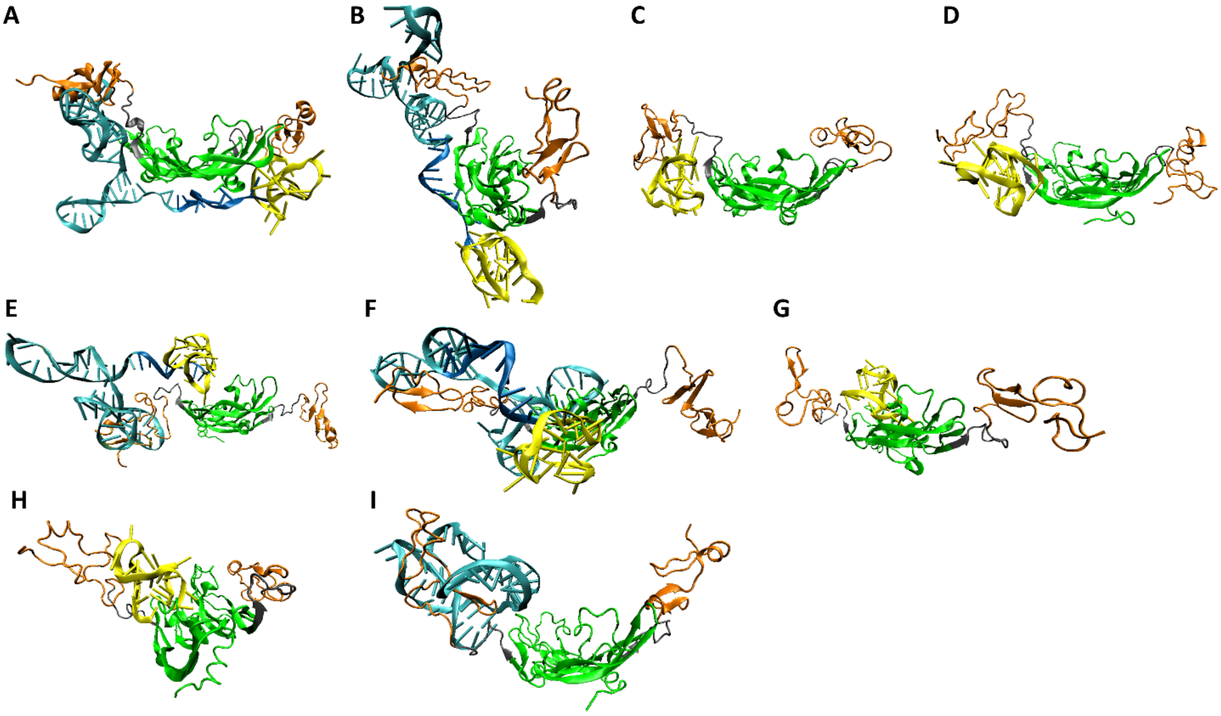

Figure S3. Side and hug poses of $VEGF_{165}$/aptamer complexes: (A) a side pose of $VEGF_{165}$/RNV66:del5-1, (B) a side pose of $VEGF_{165}$/V7t1:del5-1, (C) a side pose of $VEGF_{165}$/RNV66, (D) a side pose of $VEGF_{165}$/V7t1, (E) a hug pose of $VEGF_{165}$/RNV66:del5-1, (F) a hug pose of $VEGF_{165}$/V7t1:del5-1, (G) a hug pose of $VEGF_{165}$/RNV66, (H) a hug pose of $VEGF_{165}$/V7t1, and (I) a hug pose of $VEGF_{165}$/del5-1. No side pose was seen in the complexes of del5-1 with $VEGF_{165}$. RNV66 and V7t1 are shown in yellow and del5-1 is in cyan, with the linker between them in blue. RBD is in green and HBD is in orange with the linker between them in gray.

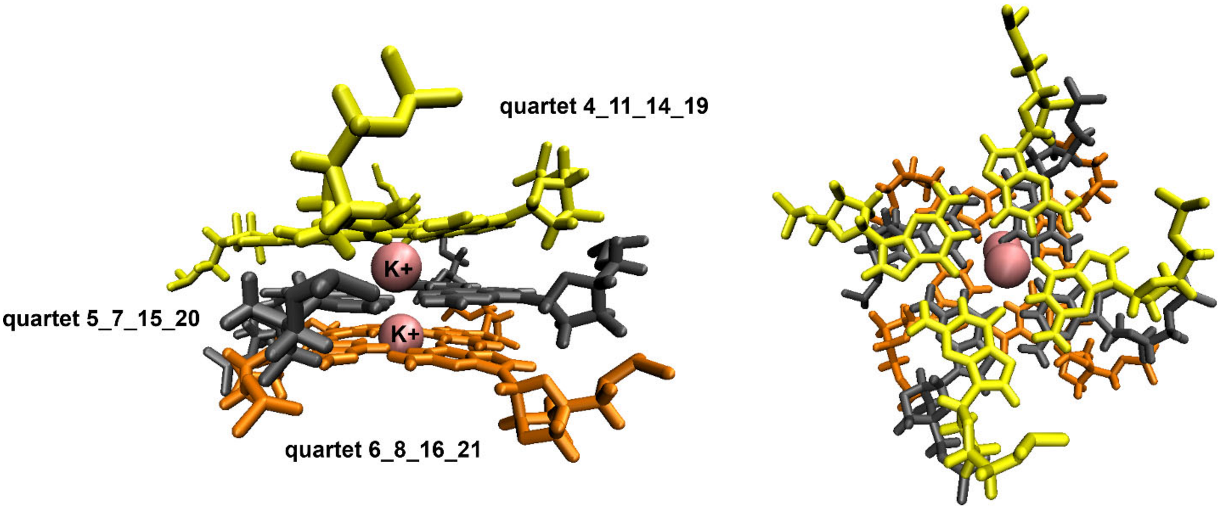

Figure S4. Schematic illustration of the G-quadruplex structure of RNV66. Three quartet planes are represented by 4-11-14-19 (yellow), 5-7-15-20 (gray), and 6-8-16-21(orange), where the numbers represent the indices of guanine nucleotides forming the G-quartet plane. The right panel shows the structure viewed from the top.

Supporting Tables

Table S1. Docking scores for $VEGF_{165}$/RNV66:del5-1 and $VEGF_{165}$/V7t1:del5-1. For each of the 84 variants composed of 12 aptamer and 7 $VEGF_{165}$ conformations, the pose with the highest docking score was selected.

Pose no.	Aptamer variant (nm)	$VEGF_{165}$ variant (nm)	Docking score (arbitrary unit)	
			$VEGF_{165}$/RNV66:del5-1	$VEGF_{165}$/V7t1:del5-1
1	2.0	3.0	−1585.66	−1332.31
2	2.0	4.0	−1406.98	−1462.43
3	2.0	5.0	−1453.60	−1500.75
4	2.0	6.0	−1661.58	−1414.71
5	2.0	7.0	−1459.98	−1371.72
6	2.0	8.0	−1419.21	−1462.75
7	2.0	9.0	−1501.44	−1379.47
8	2.5	3.0	−1376.38	−1332.15
9	2.5	4.0	−1340.17	−1314.57
10	2.5	5.0	−1394.24	−1441.49
11	2.5	6.0	−1418.07	−1459.87
12	2.5	7.0	−1491.36	−1442.13
13	2.5	8.0	−1394.94	−1323.66
14	2.5	9.0	−1460.88	−1494.86
15	3.0	3.0	−1533.36	−1358.27
16	3.0	4.0	−1467.09	−1428.88
17	3.0	5.0	−1468.25	−1446.66
18	3.0	6.0	−1490.43	−1429.68
19	3.0	7.0	−1428.29	−1409.06
20	3.0	8.0	−1483.33	−1438.75
21	3.0	9.0	−1421.65	−1520.67
22	3.5	3.0	−1359.34	−1482.53
23	3.5	4.0	−1367.87	−1509.37
24	3.5	5.0	−1411.33	−1484.80

25	3.5	6.0	−1472.44	−1525.07
26	3.5	7.0	−1426.56	−1436.94
27	3.5	8.0	−1398.79	−1484.69
28	3.5	9.0	−1426.25	−1504.26
29	4.0	3.0	−1528.69	−1357.21
30	4.0	4.0	−1495.14	−1455.48
31	4.0	5.0	−1610.47	−1574.14
32	4.0	6.0	−1464.47	−1473.17
33	4.0	7.0	−1472.31	−1473.99
34	4.0	8.0	−1450.83	−1522.74
35	4.0	9.0	−1621.94	−1413.32
36	4.5	3.0	−1426.16	−1447.03
37	4.5	4.0	−1472.15	−1397.56
38	4.5	5.0	−1493.81	−1509.98
39	4.5	6.0	−1448.33	−1446.58
40	4.5	7.0	−1583.54	−1404.61
41	4.5	8.0	−1518.56	−1380.32
42	4.5	9.0	−1531.82	−1480.95
43	5.0	3.0	−1380.37	−1335.54
44	5.0	4.0	−1382.32	−1340.19
45	5.0	5.0	−1454.60	−1585.07
46	5.0	6.0	−1496.28	−1416.07
47	5.0	7.0	−1431.18	−1464.41
48	5.0	8.0	−1305.42	−1384.47
49	5.0	9.0	−1377.28	−1386.00
50	5.5	3.0	−1636.68	−1470.79
51	5.5	4.0	−1425.01	−1379.53
52	5.5	5.0	−1329.79	−1430.80
53	5.5	6.0	−1380.04	−1347.92
54	5.5	7.0	−1349.97	−1356.03
55	5.5	8.0	−1358.41	−1459.56
56	5.5	9.0	−1347.19	−1423.12
57	6.0	3.0	−1400.87	−1474.10
58	6.0	4.0	−1323.23	−1479.43

59	6.0	5.0	−1238.59	−1362.00
60	6.0	6.0	−1384.32	−1305.59
61	6.0	7.0	−1301.92	−1491.21
62	6.0	8.0	−1287.77	−1307.84
63	6.0	9.0	−1413.63	−1300.23
64	6.5	3.0	−1438.00	−1357.71
65	6.5	4.0	−1401.28	−1273.88
66	6.5	5.0	−1376.03	−1422.60
67	6.5	6.0	−1470.37	−1313.39
68	6.5	7.0	−1344.00	−1384.46
69	6.5	8.0	−1465.69	−1342.57
70	6.5	9.0	−1358.48	−1387.27
71	7.0	3.0	−1525.94	−1342.41
72	7.0	4.0	−1298.53	−1245.42
73	7.0	5.0	−1424.56	−1368.05
74	7.0	6.0	−1413.82	−1290.63
75	7.0	7.0	−1259.39	−1322.03
76	7.0	8.0	−1335.68	−1435.95
77	7.0	9.0	−1361.75	−1368.25
78	7.5	3.0	−1260.05	−1209.06
79	7.5	4.0	−1372.79	−1294.95
80	7.5	5.0	−1297.33	−1240.71
81	7.5	6.0	−1365.60	−1378.54
82	7.5	7.0	−1227.52	−1327.40
83	7.5	8.0	−1371.70	−1286.71
84	7.5	9.0	−1320.48	−1286.48

Table S2. Docking scores for $VEGF_{165}$/RNV66, $VEGF_{165}$/V7t1, and $VEGF_{165}$/del5-1. For each of the 7 variants resulting from the 7 $VEGF_{165}$ conformations and one apatamer conformation, the top 3 poses with the highest docking scores were selected.

Pose no.	$VEGF_{165}$ variant (nm)	Docking score (arbitrary unit)		
		$VEGF_{165}$/RNV66	$VEGF_{165}$/V7t1	$VEGF_{165}$/del5-1
1	3.0	−1111.94	−1130.62	−1385.42
2	3.0	−1107.25	−1112.98	−1334.28
3	3.0	−1104.16	−1081.71	−1327.89
4	4.0	−1095.65	−1091.89	−1436.74
5	4.0	−1084.58	−1082.79	−1429.98
6	4.0	−1065.09	−1076.22	−1339.94
7	5.0	−1240.33	−1176.38	−1321.24
8	5.0	−1160.47	−1094.93	−1314.01
9	5.0	−1159.65	−1093.58	−1292.39
10	6.0	−1158.57	−1075.13	−1392.07
11	6.0	−1112.22	−1071.39	−1274.14
12	6.0	−1105.28	−1045.00	−1273.14
13	7.0	−1127.28	−1077.80	−1292.16
14	7.0	−1093.09	−1058.36	−1264.45
15	7.0	−1083.60	−1046.37	−1225.48
16	8.0	−1049.06	−1015.12	−1249.85
17	8.0	−1026.48	−994.86	−1198.54
18	8.0	−1026.01	−986.45	−1182.81
19	9.0	−1079.94	−1073.30	−1252.40
20	9.0	−999.11	−1071.73	−1223.78
21	9.0	−987.24	−1065.54	−1199.53

Table S3. Binding free energy (ΔG) values of the top 10 complex structures for each of the five types of $VEGF_{165}$/aptamer complexes.

Complex no.	ΔG (kcal/mol)				
	$VEGF_{165}$/ RNV66:del5-1	$VEGF_{165}$/ V7t1:del5-1	$VEGF_{165}$/ RNV66	$VEGF_{165}$/ V7t1	$VEGF_{165}$/ del5-1
1	−237.3	−237.2	−106.9	−152.4	−140.1
2	−234.8	−216.0	−86.4	−150.7	−137.6
3	−226.2	−209.0	−70.6	−120.3	−136.3
4	−218.5	−203.6	−69.6	−116.3	−133.5
5	−207.2	−199.0	−61.4	−116.3	−117.9
6	−205.8	−194.0	−59.1	−104.1	−114.2
7	−194.7	−190.4	−57.7	−90.1	−111.8
8	−194.6	−188.6	−56.0	−88.6	−109.0
9	−184.8	−188.3	−55.5	−88.1	−108.5
10	−184.6	−187.3	−55.2	−81.4	−94.0
Average	−208.9 ± 18.6	−201.3 ± 15.1	−67.84 ± 16.0	−110.8 ± 24.1	−120.3 ± 14.8

Table S4. Binding poses for the top ten complex structures of each $VEGF_{165}$/aptamer complex type.

Complex no.	$VEGF_{165}$/ RNV66:del5-1	$VEGF_{165}$/ V7t1:del5-1	$VEGF_{165}$/ RNV66	$VEGF_{165}$/ V7t1	$VEGF_{165}$/ del5-1
1	Sandwich	Sandwich	Sandwich	Hug	Sandwich
2	Sandwich	Sandwich	Side	Sandwich	Sandwich
3	Sandwich	Side	Hug	Sandwich	Sandwich
4	Sandwich	Sandwich	Sandwich	Side	Sandwich
5	Side	Sandwich	Sandwich	Sandwich	Sandwich
6	Sandwich	Sandwich	Sandwich	Sandwich	Sandwich
7	Sandwich	Sandwich	Sandwich	Sandwich	Sandwich
8	Side	Sandwich	Sandwich	Sandwich	Sandwich
9	Sandwich	Hug	Hug	Sandwich	Hug
10	Sandwich	Sandwich	Sandwich	Sandwich	Sandwich
